# Diagnostic Yield of CT Pulmonary Angiogram in the Diagnosis of Pulmonary Embolism and Its Predictive Factors

**DOI:** 10.7759/cureus.40484

**Published:** 2023-06-15

**Authors:** Chooi Leng Low, Ren Yi Kow, Azian Abd Aziz, Mubarak Mohd Yusof, Bee Chiu Lim, Norie Azilah Kamarudin, Ahmad Razali Md Ralib Md Raghib

**Affiliations:** 1 Department of Radiology, International Islamic University Malaysia, Kuantan, MYS; 2 Department of Orthopaedics, Traumatology and Rehabilitation, International Islamic University Malaysia, Kuantan, MYS; 3 Department of Radiology, Hospital Tengku Ampuan Afzan, Kuantan, MYS; 4 Clinical Research Centre, Hospital Tengku Ampuan Afzan, Kuantan, MYS

**Keywords:** pneumonia, covid 19, pulmonary disease, risk scoring system, predictive risk factors, computed tomography (ct ), multidetector computed tomography (ct) pulmonary angiography (ctpa), pulmonary embolism (pe)

## Abstract

Introduction

Computed tomography pulmonary angiography (CTPA) is the reference investigation of choice to diagnose pulmonary embolism (PE). Nevertheless, the use of CTPA should be weighed against its risks, such as radiation and contrast-induced nephropathy. We aim to assess the yield of CTPA in diagnosing PE at a tertiary centre in Malaysia. We also identify predictive factors associated with the yield of CTPA in this cohort.

Methods

This was a cross-sectional study involving all patients who had had CTPA done at Hospital Tengku Ampuan Afzan, Kuantan, Malaysia, from January 1, 2021, to November 30, 2021. All patients’ records were retrieved and reviewed. CTPA images were retrieved from the Radiology Information System (RIS) and Picture Archiving and Communication System (PACS). They were double-reviewed by the authors, with the initial reports redacted from reporting radiologists to prevent reporting bias. The predictive factors were determined using simple logistic regression and multiple logistic regression.

Results

A total of 351 CTPAs were reviewed, of which 93 were found to be positive for PE, giving rise to an overall CTPA yield of 26.5%. Upon simple logistic regression, factors such as gender, discipline, history of trauma, presence of COVID-19 infection, and pneumonia were found to be associated with positive CTPA. Upon multiple logistic regression, male patients were found to have a higher chance of positive CTPA results. On the other hand, patients with COVID-19 infection and pneumonia have a lower chance of positive results in CTPA.

Conclusion

The yield of CTPA in diagnosing PE at our institution was acceptable at 26.5%. Upon multiple logistic regression, patients with COVID-19 infection and pneumonia were more likely to have a negative CTPA result, highlighting the need for clinicians to be more prudent in requesting CTPAs in these patients.

## Introduction

Pulmonary embolism (PE) is clinically defined as obstruction of the pulmonary arteries by blood clots that travel via the bloodstream in the body [1-5]. It is part of venous thromboembolism, which also includes deep vein thrombosis (DVT) [1-5]. Globally, PE is attributed as the third most common cause of cardiovascular death after stroke and heart attack [1]. If it is left untreated, PE has a high mortality rate of up to 25%. Owing to the high mortality rate, PE places a tremendous amount of healthcare and economic burden on the country [1]. It is estimated that up to $10 billion is spent annually in the United States on the treatment of PE [2].

In view of the high morbidity and mortality rate associated with PE, it is important to make a prompt diagnosis and initiate anticoagulant treatment in patients with PE [3-5]. With the advancement of medical technology, computed tomography pulmonary angiography (CTPA) has become the reference standard to diagnose PE [6-10]. CTPA can be performed in patients with suspected PE within minutes, and it has high sensitivity and specificity [11-13]. The sensitivity and specificity of CTPA in diagnosing PE range between 96% and 100% and between 89% and 98%, respectively [14]. Nevertheless, the advancement of medical imaging also comes with the problem that clinicians may be over-reliant on CTPA to rule out PE [6-16]. This is evidenced by the increasing number of CTPAs ordered and the decreasing yield of CTPAs performed [6-16].

The combination of an increased number of CTPAs performed and a decreased positive rate of CTPAs not only wastes precious medical resources but also exposes patients to unnecessary radiation, especially pregnant patients [16-18]. In order to reduce the number of unnecessary CTPAs in patients with pulmonary problems, scoring systems such as Wells and Geneva Scores have been introduced [19-21]. Nonetheless, these scoring systems have not been properly adhered to, and they are not a good predictor for PE in certain patients, such as those under critical care [19-21]. Similarly, the D-dimer test is being used to rule out PE, and a negative D-dimer test is valuable in ruling out PE in high-risk patients [21-23]. Despite being a good screening test, the D-dimer test still poses some limitations as its level is age-dependent and certain conditions such as infection, trauma, and malignancy can cause its elevation in patients without PE [21-23].

Thus far, there is no consensus on the acceptable positive rate of CTPA in diagnosing PE. The Royal College of Radiologists suggests a minimum yield of 15.4% as the acceptable positive rate of CTPA, while certain centres use 10% as the accepted threshold under which overuse of CTPA should be considered [10,24]. Hitherto, there is no report on the yield of CTPA in diagnosing PE in Malaysia. Hence, our aim in this study is to investigate the yield of CTPA in diagnosing PE at the largest referral centre on the East Coast of Peninsular Malaysia. Besides that, we also investigated the predictive factors of positive CTPA scans among patients who had undergone CTPA.

## Materials and methods

This research has received ethical approval from the National Medical Research Registry of Malaysia (NMRR-21-1654-60448). Informed consent was waived owing to the cross-sectional nature of this study. This was a cross-sectional study that included all patients who had CTPA done for suspected acute PE at Hospital Tengku Ampuan Afzan, Kuantan, Pahang, Malaysia, from January 1, 2021, to November 30, 2021. No patient was excluded from this study.

All patients were identified via the electronic record system, and their demographic, clinical, and radiological data were extracted and reviewed. The following demographic information was gathered from the patient’s records: age, gender, ethnicity, requesting department, pregnancy status, presence of trauma, malignancy, COVID-19 infection, or pneumonia. A patient was deemed to have had a positive traumatic episode if the initial injury directly immobilized the patient, leading to pulmonary complications, irrespective of how long the traumatic event had been sustained. Patients were diagnosed as COVID-19 positive by reverse transcription polymerase chain reaction (RT-PCR). The diagnosis of pneumonia was made if they presented with clinical symptoms of acute infection and chest radiographs demonstrated opacities [25].

All the CTPA scans were done with a 256-slice CT scanner (SOMATOM Definition Flash, Siemens Healthineers, Erlangen, Germany) following standard protocol. The scans were performed in the pulmonary arterial phase with a 0.5 mm slice thickness reconstructed at 1.0 mm thickness, kV of 100 to 120, and auto mA. The intravenous contrast administered was a non-ionic, low-osmolar iodinated contrast medium, Iopamiro (Bracco, Milan, Italy), 300 mg iodine/mL. The volume of contrast used was 50 to 70 mL at a rate of 5 mL/second, followed by 20 mL of normal saline at the same rate. Post-processing multiplanar reconstruction was done on a dedicated workstation (Siemens SyngoVia VB10, Siemens Healthineers, Erlangen, Germany).

**Figure 1:**
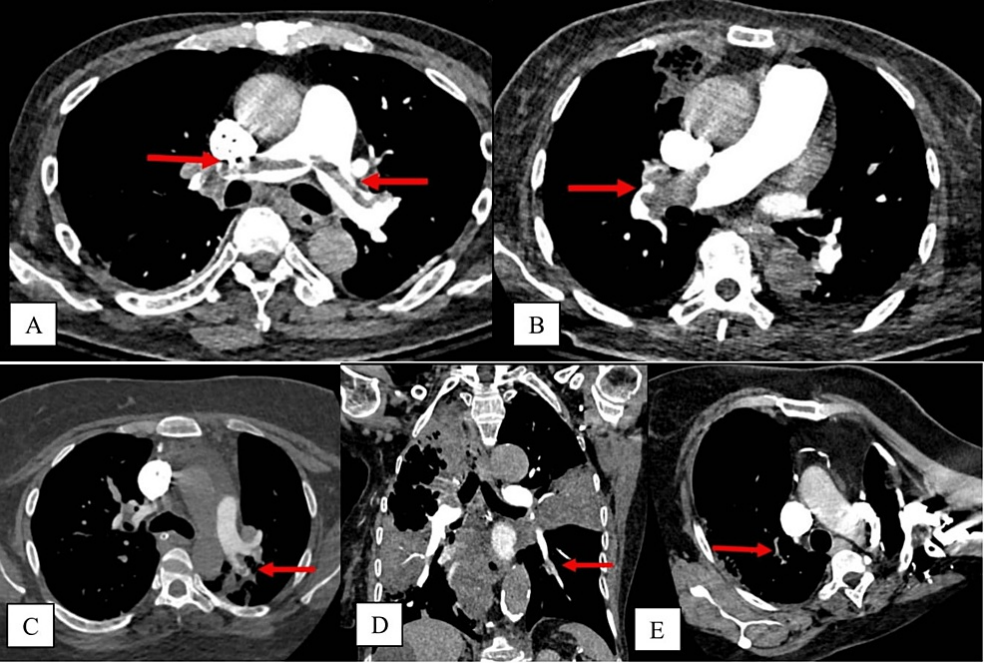
Sequential figures to illustrate the emboli at different anatomical locations (marked by red arrows). (A) Pulmonary trunk; (B) right pulmonary artery; (C) left interlobar artery; (D) left laterobasal segmental branch; (E) right upper lobe sub-segmental branch.

CTPA images were retrieved from the Radiology Information System (RIS) and Picture Archiving and Communication System (PACS). They were double-reviewed by the authors (radiologists with more than 15 years of experience). Any discrepancy between the reviewing radiologists was resolved via discussion. To prevent reporting bias, the reporting radiologists redacted the initial reports. The scans were classified as positive (presence of PE) or negative (absence of PE). There was no indeterminate or non-diagnostic CTPA image within this cohort. If PE was detected in the CTPA, the site of the most proximal extent of the embolus, whether in the pulmonary trunk, pulmonary artery, lobar/interlobar, segmental, or subsegmental arteries, was documented (Figure 1).

For the descriptive data, the continuous data were described in mean with standard deviation (SD), and the categorical data were expressed in number (n) and percentage. The yield of CTPA was defined as the ratio of the number of positive CTPAs to the total number of CTPA scans. Statistical analysis was performed with SPSS software version 20 (IBM Corp., Armonk, USA). Simple logistic regression and multiple logistic regression were performed to determine predictive factors for CTPA results within this cohort.

## Results

A total of 351 CTPAs were performed during the study period. They were performed on 351 patients, with no repeat scans. Table 1 summarized the demographic data of the patients included in this study. The mean age of patients was 50.3 years, with a standard deviation of 15.4 years. Slightly more than half were male patients (52.7%). The majority of the patients were of Malay ethnicity (90.3%). This was followed by Chinese (6.0%), others (2.3%), and Indians (1.4%). Almost four out of five CTPAs were ordered by an internal medical specialty (77.8%). This was followed by surgical specialties (10.0%), obstetrics (7.1%), orthopaedics (4.3%), and others (0.9%). Among those patients who had undergone CTPAs, 7.1% were pregnant, and 11.7% had malignancies. Eighteen patients (5.1%) had a history of trauma that had caused them to be immobilized. More than one-third of patients (37.9%) had an active COVID-19 infection, and more than one-quarter of patients (26.2%) had evidence of pneumonia.

**Table 1:** Demographic data of the patients included in this study. SD: standard deviation; O&G: obstetric and gynaecology.

Factors		Number (n)	Percentage (%)	Mean (SD)
Age		-	-	50.3 (15.4)
Gender	Male	185	52.7	-
	Female	166	47.3	-
Ethnicity	Malay	317	90.3	-
	Chinese	21	6.0	-
	Indian	5	1.4	-
	Others	8	2.3	-
Discipline	Medical	273	77.8	-
	Surgical	35	10.0	-
	O&G	25	7.1	-
	Orthopaedic	15	4.3	-
	Others	3	0.9	-
Pregnant	Yes	25	7.1	-
	No	326	92.9	-
Trauma	Yes	18	5.1	-
	No	333	94.9	-
Malignancy	Yes	41	11.7	-
	No	310	88.3	-
COVID-19 infection	Yes	133	37.9	-
	No	218	62.1	-
Pneumonia	Yes	92	26.2	-
	No	259	73.8	-

Among the 351 CTPAs performed within the study period, 93 of them (26.5%) had evidence of PE (Table 2). Half of those (n=47, 50.5%) were found within segmental pulmonary arteries. This was followed by the pulmonary artery (n=18, 19.4%), subsegmental (n=17, 18.3%), lobar/interlobar (n=8, 8.6%), and pulmonary trunk (n=3, 3.2%). Most of the PEs were present on the right side (n=42, 45.2%), followed by bilateral (n=32, 34.4%) and the left side (n=19, 20.4%). On simple logistic regression, factors such as gender, discipline, trauma, COVID-19 infection, and pneumonia were found to be associated with a positive CTPA (Table 3). Nonetheless, after multiple logistic regression, only factors such as gender, COVID-19 infection, and pneumonia were found to be predictive factors for CTPA. Male patients were found to be positively predictive for the presence of PE, whereas patients with COVID-19 infection and pneumonia were more likely to have a negative CTPA result (Table 3).

**Table 2:** Results of the computed tomographic pulmonary angiogram included in this study. CTPA: computed tomographic pulmonary angiogram, PE: pulmonary embolism.

CTPA results		Number (n)	Percentage (%)
Presence of PE	Yes	93	26.5
	No	258	73.5
Side	Right	42	45.2
	Left	19	20.4
	Bilateral	32	34.4
Location	Sub-segmental	17	18.3
	Segmental	47	50.5
	Lobar/interlobar	8	8.6
	Pulmonary artery	18	19.4
	Pulmonary trunk	3	3.2

**Table 3:** Analysis via simple logistic regression and multiple logistic regression to determine the predictive factors of the yield of computed tomographic pulmonary angiogram. Nil: not available, OR: odd ratio, b: regression coefficient, aForward LR multiple logistic regression was applied. Multicollinearity and interaction were checked and not found. Hosmer-Lemeshow test, (p-value = 0.486), Pearson chi-square test, (p-value =0.325), classification tale (overall correctly classified percentage = 75.8%), and area under the ROC curve (70.9%) were applied to check the model fit.

Variables	Simple logistic regression			Multiple logistic regression		
	b	Crude OR (95% CI)	p-value	b	Adjusted OR (95% CI)	p-value
Age	−0.003	0.997 (0.982, 1.012)	0.680			
Gender						
Female (ref)	0	1.00		0	1.00	
Male	0.723	2.060 (1.259, 3.371)	0.004	0.804	2.235 (1.328, 3.760)	0.002
Ethnicity						
Malay (ref)	0	1.00				
Chinese	0.535	1.707 (0.683, 4.263)	0.252			
Indian	−20.183	Nil (Nil, Nil)	>0.950			
Others	−0.926	0.396 (0.048, 3.269)	0.390			
Discipline						
Medical (ref)	0	1.00				
Surgical	0.896	2.449 (1.185, 5.060)	0.016			
O&G	0.608	1.837 (0.775, 4.355)	0.167			
Ortho	0.490	1.633 (0.538, 4.952)	0.386			
Others	−20.019	0 (0, Nil)	>0.950			
Pregnancy						
No (ref)	0	1.00				
Yes	0.082	1.085 (0.438, 2.688)	0.860			
Trauma						
No (ref)	0	1.00				
Yes	1.087	2.964 (1.139, 7.714)	0.026			
Malignancy						
No (ref)	0	1.00				
Yes	0.661	1.937 (0.983, 3.816)	0.056			
Covid						
No (ref)	0	1.00		0	1.00	
Yes	−0.743	0.475 (0.281, 0.805)	0.006	−1.260	0.284 (0.160, 0.503)	<0.001
Non-covid pneumonia						
No (ref)	0	1.00		0	1.00	
Yes	−0.999	0.368 (0.194, 0.700)	0.002	−1.652	0.192 (0.096, 0.384)	<0.001

## Discussion

In the United States, it is estimated that 2.4 million CTPA scans are performed each year to look for PE in the emergency department [15]. The number of CTPAs is predicted to increase worldwide due to the increasing availability of computed tomography machines in many parts of the world [3,6]. Despite being the gold standard for diagnosing PE, CTPA is not without risk. It is associated with a median radiation dose of 10 mSV, equivalent to 137 plain radiographs of the chest [15]. Unjustified CTPAs expose patients to unnecessary radiation, endangering them with the stochastic risk of radiation-induced malignancy [15]. Besides exposing patients to the risks of radiation, CTPA also carries potential adverse effects from intravenous contrast administration, such as contrast-induced nephropathy [11]. For each CTPA performed, a patient is exposed to a risk of up to 14% of contrast-induced nephropathy and a lifetime malignancy risk of 2.8% [15]. Hence, it is important for the clinician to evaluate the risk of PE in a patient prior to subjecting the patient to a CTPA.

There is a wide range of diagnostic yields for CTPA for PE in the literature, ranging from 4.7% to 31% [3-12]. Despite using a lower cut-off (10%) as the acceptable threshold in North America, there are a few studies that report a yield lower than the threshold [3-12]. In Malaysia, we adopt the threshold of 15.4% as the acceptable rate for the yield of CTPA, as recommended by the Royal College of Radiologists [10,24]. The yield of CTPA in this cohort is acceptable at a rate of 26.5%. Among the 93 patients who are diagnosed with PE on CTPAs, more than 80% of these patients have PE at segmental pulmonary arteries or more proximal levels. Up to one-fifth of patients (19.4%) have PE within the main pulmonary arteries. Besides that, one-third of patients (34.4%) have PE bilaterally. These results indicate the severity of the emboli affecting these patients.

In this cohort, we also investigate factors predicting the outcomes of CTPA. On multiple logistic regression, we find that male patients have a higher chance of positive CTPA results. This finding is consistent with the study by Kindermann et al., which also reported that male patients tend to have a higher risk of PE [26]. This may be partially due to the fact that male patients are associated with an increased risk of venous thromboembolism [15,26]. Additionally, the late presentation may have an impact on the higher positive yield of CTPA in male patients because they frequently present with more severe clinical symptoms, increasing the likelihood of detecting a PE in CTPA.

Patients with COVID-19 infection and pneumonia are found to have a lower chance of positive CTPA results. The study period in this cohort may have been the factor leading to this finding. The study is conducted at a time when several COVID-19-related studies are published, demonstrating that patients with COVID-19 infections are at increased risk of developing thrombosis [27-29]. We postulate that these studies may have influenced treating physicians to proceed with CTPA even though there is a lack of clinical evidence suggesting PE, lowering the odds of positive results in these CTPAs.

Similar to patients with COVID-19 infection, patients with clinical and radiological signs of pneumonia are less likely to have PE on CTPA. Even though both PE and pneumonia presented with clinical signs suggestive of pulmonary diseases, and sometimes they are difficult to differentiate, we found that patients with patchy opacities or ground-glass changes on plain radiographs were less likely to have PE on CTPA. To the best of our knowledge, this study is the first to show the negative predictive value of COVID-19 infection and pneumonia in the yield of CTPA.

Limitation

There are several limitations to this study. First, a relatively small sample size from a single centre makes generalization of the findings impossible. Second, the cross-sectional nature of this study and lack of proper adherence to predictive scoring systems preclude several important parameters, such as clinical signs and D-dimer results, hence making a comparison of scoring systems such as the Geneva score or Well’s score difficult [15].

## Conclusions

The yield of CTPA in diagnosing PE at our institution is acceptable at 26.5%. Among patients who are diagnosed with PE on CTPAs, one-third of them have PE bilaterally, and more than 80% of them have PE at segmental pulmonary arteries or more proximal levels, highlighting the severity of the emboli affecting these patients. On multiple logistic regression, patients with COVID-19 infection and pneumonia are more likely to have a negative CTPA, highlighting the need for clinicians to be more prudent in requesting CTPAs in these patients.
